# Pathogenic *Leptospira* spp. in Wild Rodents, Canary Islands, Spain

**DOI:** 10.3201/eid1709.101470

**Published:** 2011-09

**Authors:** Pilar Foronda, Aarón Martin-Alonso, Borja del Castillo-Figueruelo, Carlos Feliu, Horacio Gil, Basilio Valladares

**Affiliations:** Author affiliations: University of La Laguna, Canary Islands, Spain (P. Foronda, A. Martin-Alonso, B. del Castillo-Figueruelo, B. Valladares);; University of Barcelona, Barcelona, Spain (C. Feliu);; Instituto de Salud Carlos III, Madrid, Spain (H. Gil)

**Keywords:** pathogenic Leptospira, leptospirosis, Rattus rattus, Mus domesticus, rats, mice, wild rodents, Canary Islands, bacteria, Spain, zoonotic diseases, zoonoses, letter

**To the Editor:** Leptospirosis is a major emerging infectious disease with a worldwide distribution (*1*). It is a systemic disease of humans and domestic animals (*2*). Regarded globally as a zoonosis because it is acquired by humans from contact with animals or from water contaminated with the urine of infected animals, it is presumed to be the most widespread zoonotic disease in the world (*1**,**2*). Species such as mice (*Mus* spp.) and rats (mainly *Rattus norvegicus* and *R. rattus*) serve as reservoirs for their host-related serovars (*3*).

Human patients usually exhibit a nonspecific self-limiting febrile illness; however, in 5%–10% of cases, severe forms of the disease develop, including Weil disease and severe pulmonary hemorrhagic syndrome. Case-fatality rates for Weil disease and severe pulmonary hemorrhagic syndrome are >10% and >74%, respectively (*4**,**5*).

Because leptospirosis has been found in humans in the Canary Islands (J. Alcoba-Flores, pers. comm.), detecting carrier animals is vital to the understanding of enzootic and epizootic leptospirosis in this environment. We examined the possible role of the rodent species found in the Canary Islands in the transmission of this pathogen to determine the risk for humans in these islands.

A total of 149 wild rodents (74 *R. rattus* and 75 *Mus domesticus*) were captured during 20009–2010 from 4 of the Canary Islands (Tenerife, El Hierro, La Gomera, Lanzarote). Urinary bladders of the animals were collected and preserved in 100% ethanol. Genomic DNA was extracted by using the Fast DNA Kit (BIO 101 Systems: MP Biomedicals, Santa Ana, CA, USA).

The *lipL32* fragment (497 bp), which is present only in pathogenic leptospires, was amplified according to the method of Bomfim et al. (*6*) by using a MyCycler thermocycler (Bio-Rad, Hercules, CA, USA). *L. interrogans* serovar Icterohemorragiae (RGA strain) was used as a positive control.

Twenty-two samples were positive for *Leptospira* spp., indicating a general prevalence of 14.8% in the rodents. Although the prevalence was higher in rats (20.3%) than in mice (9.3%) (Table), the difference was not significant (χ^2^ test). Positive samples were obtained from all the studied islands and for both host species in all of them (Table), without significant differences in the prevalences between host species or between islands.

**Table:** Prevalence of pathogenic *Leptospira* spp. in rodents, by island, Canary Islands, 2009–2010*

Island and host species	No. (% positive)	% Prevalence (95% CI)
Tenerife	11 (49)	22.4 (10.7–34.1)
* Mus domesticus*	2 (12)	16.6 (0–37.6)
* Rattus rattus*	9 (37)	24.3 (10.5–38.1)
La Gomera	4 (16)	25.0 (3.8–46.2)
* M. domesticus*	3 (10)	30.0 (1.6–58.4)
* R. rattus*	1 (6)	16.7 (0–46.5)
El Hierro	2 (29)	6.9 (0–16.1)
* M. domesticus*	1 (16)	6.2 (0–18.11)
* R. rattus*	1 (13)	7.7 (0–22.2)
Lanzarote	5 (55)	9.1 (1.5–16.7)
* M. domesticus*	1 (37)	2.7 (0–7.9)
* R. rattus*	4 (18)	22.2 (3–41.4)
Total	22 (149)	14.8 (11.9–17.7)
* M. domesticus*	7 (75)	9.3 (2.7–15.9)
* R. rattus*	15 (74)	20.3 (11.1–29.5)

*No., number of rodents studied; positive, samples positive for pathogenic *Leptospira* spp.; CI, confidence interval.

To confirm the amplified products belonged to pathogenic *Leptospira* spp., we sequenced some amplicons. Sequencing reactions were performed for both strands at the University of La Laguna Genomic Service. When the sequences were compared, 2 different sequences were obtained. The first sequence, L19 (GenBank accession no. HQ231747), from rats, clustered with *L. interrogans* serovar Copenhageni (GenBank accession no. AE016823) and different serovars of the same species by BLAST (99% identity). Previous results associate *L. interrogans* serovar Copenhageni with *Rattus* spp (*7*). However, the sequence obtained from mice (GenBank accession no. HQ231748), L47, showed a 100% BLAST identity with *L. borgpetersenii* (GenBank accession nos. DQ320625.1 and DQ286415.1).

New and published *Leptospira* sequences were aligned with the multiple alignment program ClustalW in MEGA3.1 (*8*), and minor corrections were made manually. The alignment for the 497-bp fragment starts at nt position 208, with respect to the complete sequence of the *lipL32* (AY609332), and ends at nt position 705.

Phylogenetic relationships were inferred by using the neighbor-joining distance method with MEGA3.1. At least 1,000 bootstrap replicates were used to infer statistical support at branch nodes. The consensus tree yielded 3 monophyletic groups clearly separated by high bootstrap values. The first clade was formed by *L. interrogans, L. kirschneri,* and *L. noguchii* (93% bootstrap value). The sequence L19 was included in the *L. interrogans* node (92% bootstrap value). The second clade included *L. borgpetersenii* and *L. weilii* as a monophyletic group (97% bootstrap value). The sequence L47 clustered with *L. borgpetersenii* DQ286415 with a high bootstrap value (82%). These results are in accordance with those obtained by Haake et al. (*9*) based on *lipL32.* Finally, *L. santarosai* sequences formed the third separate clade (100% bootstrap) (data not shown).

Although the method we used does not enable specific identification, determining the most similar species by BLAST is needed for control programs. *L. interrogans* serovar Copenhageni is the predominant infecting serovar among patients with severe leptospirosis (*7*), and *L. borgpetersenii* is also commonly acquired from mice.

On the basis of these findings, the global distribution of *Leptospira* spp. must be revised to include the Canary Islands, with rodents as natural hosts. Because pathogenic *Leptospira* spp. were detected on every island studied and in both analyzed species, *R. rattus* and *M. domesticus*, the distribution of this pathogen likely extends to even the islands not studied. The high incidence found suggests that rodents play a role in transmission of human leptospirosis. Further studies are needed to identify other possible reservoir hosts and to determine the risk areas for acquiring pathogenic leptospires in the Canary Islands.

## References

[1] Levett PN. Leptospirosis. Clin Microbiol Rev. 2001;14:296–326. 10.1128/CMR.14.2.296-326.2001 11292640 PMC88975

[2] Adler B, de la Pena Moctezuma A. *Leptospira* and leptospirosis. Vet Microbiol. 2010;140:287–96. 10.1016/j.vetmic.2009.03.012 19345023

[3] Bharti AR, Nally JE, Ricaldi JN, Matthias MA, Diaz MM, Lovett MA, Leptospirosis: a zoonotic disease of global importance. Lancet Infect Dis. 2003;3:757–71. 10.1016/S1473-3099(03)00830-2 14652202

[4] McBride AJ, Athanazio DA, Reis MG. Ko Al. Leptospirosis. Curr Opin Infect Dis. 2005;18:376–86. 10.1097/01.qco.0000178824.05715.2c 16148523

[5] Gouveia EL, Metcalfe J, de Carvalho AL, Aires TS, Villasboas-Bisneto JC, Queirroz A, Leptospirosis-associated severe pulmonary hemorrhagic syndrome, Salvador, Brazil. Emerg Infect Dis. 2008;14:505–8. 10.3201/eid1403.071064 18325275 PMC2570821

[6] Bomfim MRQ, Barbosa-Stancioli EF, Koury MC. Detection of pathogenic leptospires in urine from naturally infected cattle by nested PCR. Vet J. 2008;178:251–6. 10.1016/j.tvjl.2007.07.029 17869555

[7] de Faria MT, Calderwood MS, Athanazio DA, McBride AJ, Hartskeerl RA, Pereira MM, Carriage of *Leptospira interrogans* among domestic rats from an urban setting highly endemic for leptospirosis in Brazil. Acta Trop. 2008;108:1–5. 10.1016/j.actatropica.2008.07.005 18721789 PMC2596941

[8] Kumar S, Tamura K, Nei M. MEGA3: integrated software for Molecular Evolutionary Genetics Analysis and sequence alignment. Brief Bioinform. 2004;5:150–63. 10.1093/bib/5.2.150 15260895

[9] Haake DA, Suchard MA, Kelley MM, Dundoo M, Alt DP, Zuerner RL. Molecular evolution and mosaicism of leptospiral outer membrane proteins involves horizontal DNA transfer. J Bacteriol. 2004;186:2818–28. 10.1128/JB.186.9.2818-2828.2004 15090524 PMC387810

